# Trehalose Analogues: Latest Insights in Properties and Biocatalytic Production

**DOI:** 10.3390/ijms160613729

**Published:** 2015-06-15

**Authors:** Maarten Walmagh, Renfei Zhao, Tom Desmet

**Affiliations:** Center for Industrial Biotechnology and Biocatalysis, Faculty of Bioscience Engineering, Ghent University, Coupure Links 653, Ghent 9000, Belgium; E-Mails: maarten.walmagh@ugent.be (M.W.); renfei.zhao@ugent.be (R.Z.)

**Keywords:** prebiotic, galactosidase, glycosyltransferase, glycoside phosphorylase, enzyme engineering

## Abstract

Trehalose (α-d-glucopyranosyl α-d-glucopyranoside) is a non-reducing sugar with unique stabilizing properties due to its symmetrical, low energy structure consisting of two 1,1-anomerically bound glucose moieties. Many applications of this beneficial sugar have been reported in the novel food (nutricals), medical, pharmaceutical and cosmetic industries. Trehalose analogues, like lactotrehalose (α-d-glucopyranosyl α-d-galactopyranoside) or galactotrehalose (α-d-galactopyranosyl α-d-galactopyranoside), offer similar benefits as trehalose, but show additional features such as prebiotic or low-calorie sweetener due to their resistance against hydrolysis during digestion. Unfortunately, large-scale chemical production processes for trehalose analogues are not readily available at the moment due to the lack of efficient synthesis methods. Most of the procedures reported in literature suffer from low yields, elevated costs and are far from environmentally friendly. “Greener” alternatives found in the biocatalysis field, including galactosidases, trehalose phosphorylases and TreT-type trehalose synthases are suggested as primary candidates for trehalose analogue production instead. Significant progress has been made in the last decade to turn these into highly efficient biocatalysts and to broaden the variety of useful donor and acceptor sugars. In this review, we aim to provide an overview of the latest insights and future perspectives in trehalose analogue chemistry, applications and production pathways with emphasis on biocatalysis.

## 1. Introduction

**Figure 1:**
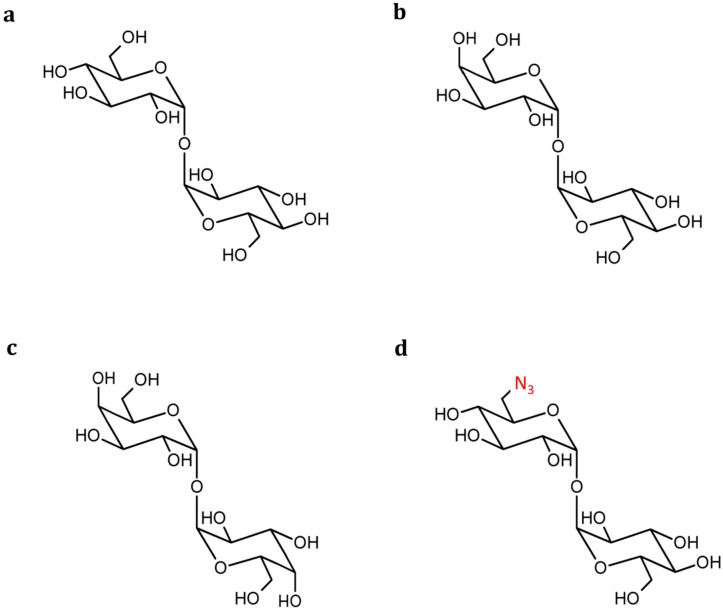
Structure of trehalose (**a**) and its analogues lactotrehalose (**b**); galactotrehalose (**c**); and 6-azidotrehalose (**d**). The azide-functional group is indicated in red.

Trehalose (α-d-glucopyranosyl α-d-glucopyranoside or α,α-trehalose) is a non-reducing disaccharide that consists of two 1,1-linked α-glucose monosaccharides (Figure 1a) and possesses interesting properties for the food and pharmaceutical industry [1]. In nature, it is present as osmolyte in a wide range of organisms, most notably yeasts and plants, where it protects the host against environmental stresses. In addition, trehalose is also stable at a wide range of pH-values, has a mild sweet taste and is not cariogenic. These features make it ideally suited for use in processed food, where trehalose is now even the “functional oligosaccharide” with the highest market share [2]. The downside is that ingested trehalose also contributes to the caloric intake as it can be efficiently hydrolysed in the small intestine by trehalases (α,α-trehalose-1-*C*-glucohydrolase, EC (Enzyme Commission) number 3.2.1.28), present in the intestinal villi, and then absorbed in the form of glucose [3].

Analogues of trehalose containing other carbohydrate moieties besides glucose are believed to have a more beneficial metabolic profile. For example, lactotrehalose (α-d-galactopyranosyl α-d-glucopyranoside), where the first glucose unit has been replaced by galactose (Figure 1b), is not a substrate for intestinal trehalase but functions as a competitive inhibitor of the same enzyme [4]. Consequently, this disaccharide does not contribute to the energy content of food preparations and at the same time also lowers the metabolic conversion of trehalose. A crucial factor is that it still offers the same stabilizing properties as trehalose by virtue of the non-reducing α-(1,1)-α linkage [5].

In addition to their benefits for the food industry, trehalose analogues also show properties that are of interest to the pharmaceutical field. As trehalose is, unlike in humans, an essential metabolite for *Mycobacteria*, deoxy- or azido-derivatives of trehalose (Figure 1d) form valuable tools to inhibit mycobacterial cell growth or for future imaging and detection of *Mycobacterium tuberculosis*, the causative agent of tuberculosis [6].

Despite their value, analogues of trehalose have not yet been studied very extensively because no cost-effective process for their large-scale synthesis is currently available. In industry, trehalose is produced from starch through a two-step enzymatic process that makes use of the consecutive action of a transglycosidase and a glycosidase [7]. Since this basically comes down to the rearrangement of glucose molecules, other types of monosaccharides cannot be incorporated through this process. Therefore, alternative production routes for trehalose analogues have recently been explored, which will be extensively discussed and compared in this review.

## 2. Trehalose and Its Analogues: Diversity, Function and Applications

Trehalose, a known osmolyte, is relatively widespread in nature as it can be found in the nucleocytosolic compartment of bacteria, yeasts, fungi, insects, invertebrate animals and plants, but is absent in higher vertebrates. Naturally occurring trehalose analogues, or more specifically trehalose-based oligosaccharides, are less prevalent with few compounds reported in bacteria, mostly in *Mycobacteria*, and very recently in the ascospores of the fungus *Neosartorya fischeri* [8]. A galactose-containing analogue was found in *Mycobacterium smegmatis*, whereas mannopyranosyl-substituted trehaloses were isolated from *Mycobacterium bovis* BCG [9]. Two amino-analogues, namely GlcNAc-α-(1,1)-α-Glc and GlcNAc-α-(1,1)-α-Man, can be produced by certain strains of *Streptomyces* and possess antibiotic activity [10,11]. Ascospores of *Neosartorya fischeri* contain a series of trehalose-based tri-, tetra- and pentaoligosaccharides, of which the latter two, named neosartose and fischerose, respectively, have not been reported before in nature [8].

Trehalose is a versatile component that fulfills diverse biochemical functions ranging from carbon or energy source (glucose release upon hydrolysis), signaling molecule, component of cell wall glycolipids to, most importantly, protecting agent against various environmental stress conditions, including heat, freezing, reactive oxygen species and drought [12]. When faced with stress, trehalose gradually accumulates inside the cytoplasm of the organism as direct response where it prevents macromolecules from denaturing and, in addition, suppresses their aggregation [13]. These stabilizing effects of trehalose on cells and macromolecules (proteins, membranes,) have mainly been attributed to the clam shell conformation in which axial-axial-linked α-(1,1)-α disaccharides crystallize [5]. Other saccharides that crystallize in an axial-equatorial fashion, like α,β-trehalose or in a more open conformation, like sucrose, maltose and lactose, do not exert such beneficial stabilization properties. It is believed that trehalose analogues, however, should possess the same stabilizing properties of trehalose as this 1,1-linkage in an axial-axial fashion, claimed to be the key for anhydrobiotic preservation, is retained here. This proved to be the case for α,α-galactotrehalose (α-d-galactopyranosyl α-d-galactopyranoside) (Figure 1c), albeit to a smaller extent than trehalose [5]. In addition, both trehalose and its analogues are non-reducing disaccharides and as such do not engage in Maillard reactions. Trehalose itself is also remarkably stable in a wide pH range (between 3.5 and 10) and at high temperatures (up to 120 °C for 90 min) [1]. Its strong resistance to hydrolysis can be explained by the remarkably low energy content of its glycosidic bond (1 kcal/mol), when compared to that of sucrose (27 kcal/mol), for example [14]. Due to this reason, trehalose analogues are expected to possess similar resistance properties.

Thanks to its stability-enhancing features, trehalose is useful for various applications in the fields of food production, cosmetics and pharmaceuticals. It is present in many natural food products and received the GRAS (Generally Recognized As Safe) status as food additive in the year 2000. It has a less pronounced (only 45% relative sweetness) but longer lasting sweetness than sucrose [15], and can suppress the bitterness or enhance sourness of food [16]. Interestingly, trehalose also inhibits lipid oxidation by heating and exposure to air [1], thus increasing shelf-life and minimizing the release of unpleasant odors. The addition of trehalose prior to drying of fruits or vegetables prevents discoloration and stabilizes their aroma [17,18]. Despite its food stabilizing benefits, trehalose is prone to enzymatic digestion in the human gut and as such contributes to the caloric intake. Lactotrehalose (α-d-glucopyranosyl α-d-galactopyranoside), in contrast, cannot be hydrolyzed by trehalase and even showed to competitively inhibit this enzyme and other intestinal brush-border disaccharidases from rat, including sucrase, maltase and isomaltase [4]. This interesting observation opens up a myriad of possible applications, the most obvious one being the use as a low-calorie sweetener, suitable for consumption by diabetic patients. In addition, higher systemic concentrations of lactotrehalose can be reached upon oral ingestion, which should allow this analogue to outperform trehalose in therapeutic applications.

Furthermore, as lactotrehalose is not hydrolyzed in the intestines, it reaches the colon in an intact state where it could exert prebiotic properties. Indeed, di-, tri- or tetrasaccharides containing α-galactosyl, α-glucosyl and α-fructosyl residues are known to selectively stimulate the growth of health-promoting bifidobacteria [19]. Biochemical or medicinal interest may arise from the α-galactoside epitope, which is widely expressed in mammalian tissues in the form of the cell surface glycolipids or glycoproteins [20]. This epitope induces specific transfer of drugs towards the liver. Recently, lactotrehalose was proposed as a molecular mimic of globosyl (Gb) disaccharide [21]. Lactotrehalose could in this case be used to create compounds with certain beneficial properties. For example, conjugation of lactotrehalose to a Gb3 ceramide-mimetic polymer allowed detoxification and neutralization of Shiga-toxin, the major virulence factor of enterohemorrhagic *E. coli* (EHEC), which leads to severe symptoms and even death upon infection [20,22]. The inhibition of trehalase makes this compound also an interesting target for the development of environmentally friendly insecticides, as trehalose is the vital energy source for the flight of insects [23].

**Figure 2:**
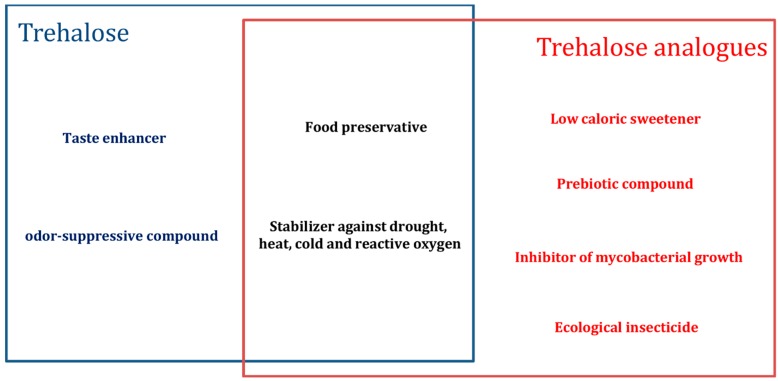
Summary of applications for trehalose and its analogues in food, pharmaceutical, cosmetics and agricultural industries.

Finally, trehalose analogues form valuable tools for probing of *Mycobacteria* via their trehalose metabolism and as potent inhibitors of mycobacterial growth [6]. These types of organisms rely on trehalose as precursor for essential cell wall glycolipids and other metabolites [24]. In the past, the trehalose metabolism was found to be related to the virulence of pathogenic mycobacteria, including *Mycobacterium tuberculosis*, the causative agent of tuberculosis [25]. Trehalose analogues chemically modified with fluorescent detectable tags, like fluorescein and azide, were successfully applied for detection and imaging of living *Mycobacteria* [6,26]. This observation is exciting as it paves the pathway for future application of tagged trehalose analogues in tuberculosis diagnostics. Furthermore, some trehalose analogues disrupt essential metabolic pathways and as such offer potential as antimycobacterial compounds [27,28]. For example, 6-azido-6-deoxy-α,α′-trehalose (Figure 1d) has the capacity to interfere with cell wall synthesis of *Mycobacterium aurum* and in this way inhibits its growth and survival [29]. Other unnatural analogues like trehalose mono-esters possess immunoregulatory properties [30], which render them very useful as adjuvant in vaccine development against tuberculosis [31]. An overview of the most valuable applications of trehalose and its analogues is provided in Figure 2.

## 3. Biocatalytic Synthesis Routes for Trehalose Analogues

**Figure 3:**
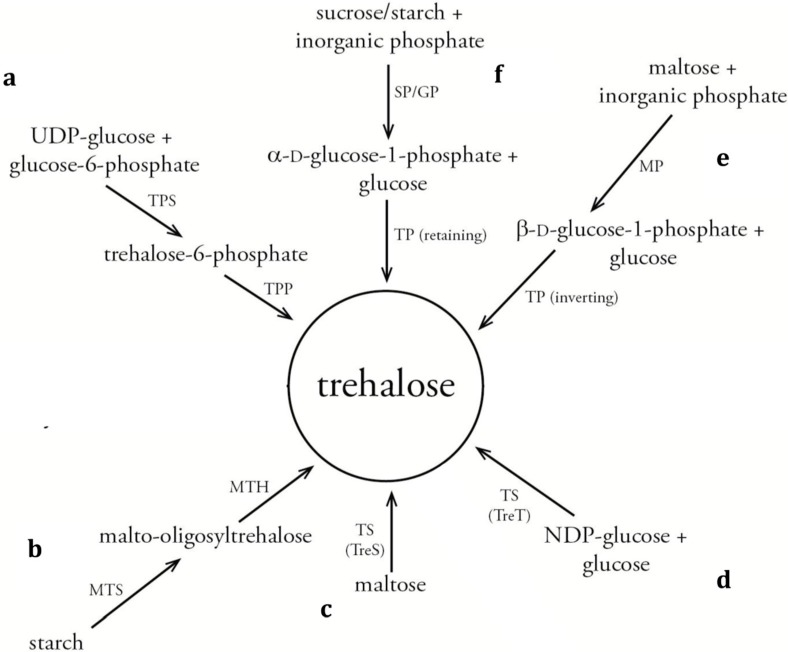
Schematic representation of biocatalytic pathways for trehalose synthesis. The traditional trehalose phosphate synthase/phosphorylase (TPS/TPP) route (**a**); the malto-oligosyltrehalose synthase/hydrolase (MTS/MTH)-coupled route (**b**); the TreS-type trehalose synthase route (**c**); the TreT-like trehalose synthase route (**d**); and the inverting (**e**) or retaining (**f**) trehalose phosphorylase (TP) routes coupled with other glycoside phosphorylases, *i.e.*, maltose phosphorylase (MP) and sucrose phosphorylase (SP), respectively. The latter three pathways (**d**–**f**) are also amenable to trehalose analogue synthesis. NDP = nucleoside diphosphate.

The interest in trehalose analogue synthesis arose in the 1970s, when the first chemical [32] and enzymatic [33] procedures were published, with even new developments being reported up to date [20,34]. In general, trehalose analogues are difficult to produce via chemical methods as these struggle to obtain C2-symmetry or to achieve a strict α-(1,1)-α anomeric control. Although some purely chemical methods have been developed for trehalose desymmetrization or for manipulation of regioselective hydroxyl-groups, these are usually lengthy, inefficient and low-yielding [9]. Furthermore, the use of heavy metal catalysts and solvents in chemical synthesis is less desirable for an end product that is expected to be used as food additive. A more “ecological” or “greener” alternative in the form of enzymatic synthesis reactions is thus highly anticipated. To that end, inspiration can be drawn from the biocatalytic synthesis of trehalose, a topic that has been studied quite intensively during the last decades [35,36,37,38]. So far, five main pathways have been described in literature for the biocatalytic production of trehalose (see Figure 3). The traditional and most widespread production method consists of two consecutive enzyme-catalyzed steps via trehalose-6-phosphate synthase (TPS, EC 2.4.1.15) and trehalose-6-phosphate phosphatase (TPP, EC 3.1.3.12) starting from uridine diphosphate (UDP)-glucose and glucose-6-phosphate with intermediary trehalose-6-phosphate formation (Figure 3a) [37]. Later on, also the malto-oligosyltrehalose synthase (MTS, EC 5.4.99.15)/malto-oligosyltrehalose hydrolase (MTH, EC 3.2.1.141) system generating trehalose out of starch [7,39] (Figure 3b) and the TreS-type trehalose synthase (EC 5.4.99.16) catalyzing the intramolecular rearrangement of maltose (α-(1,4) bond) to trehalose (α-(1,1) bond) [40,41] (Figure 3c), were acknowledged as alternative, highly efficient trehalose production systems.

Out of these pathways, only the routes using inverting or retaining trehalose phosphorylases (TP) [33,42] (Figure 3e,f) and TreT-type trehalose synthases (Figure 3d) [34,43] can be easily adapted towards biocatalytic trehalose analogue synthesis. A third possible enzymatic pathway for analogues production, not applicable for trehalose synthesis, makes use of galactosidases [44,45]. Each of these three routes comes with its own advantages and disadvantages, inherent to the respective enzyme class employed, on which will be further elaborated in this review.

### 3.1. Galactosidase-Catalyzed Production

**Figure 4:**
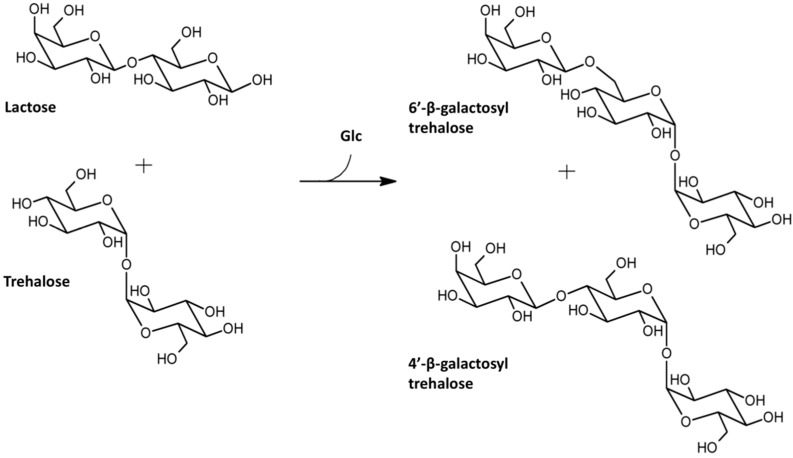
Pathway for synthesis of trehalose trisaccharide analogues via *E. coli* β-galactosidase activity starting from the disaccharides lactose and trehalose.

Galactosidases were the first enzymes ever to be exploited for the synthesis of trehalose di- or trisaccharide analogues. In 1995, a thermostable α-galactosidase (EC 3.2.1.22) from *Candida guilliermondii* was reported to generate galactotrehalose through reverse hydrolysis when high concentrations of galactose were used as substrate [44]. Unfortunately, the enzyme was not very selective and preferably formed a 1,6-linkage, with 1,3- and 1,2-isomers being formed as well. Later on, an *E. coli* β-galactosidase (EC 3.2.1.23) was used for the conversion of trehalose into trisaccharides via a transgalactosylation reaction [45]. In addition to hydrolyzing lactose, the latter enzyme was also able to catalyze the transfer of a β-galactosyl group from lactose to the 4′- as well as the 6′-position of one of the glucose moieties, yielding a mixture of 4′- and 6′-β-d-galactopyranosyl trehalose (Figure 4).

Both types of galactosidases clearly suffer from one of the typical drawbacks when using hydrolases for synthetic purposes, *i.e.*, low regioselectivity. In case the acceptor contains more than one hydroxyl-group, a mixture of products is formed, which is difficult to separate and significantly complicates downstream processing [46]. Another drawback is their low product yield, although this could be improved by controlling the reaction kinetically or thermodynamically [47].

### 3.2. TreT-Type Trehalose Synthase-Catalyzed Production

**Figure 5:**
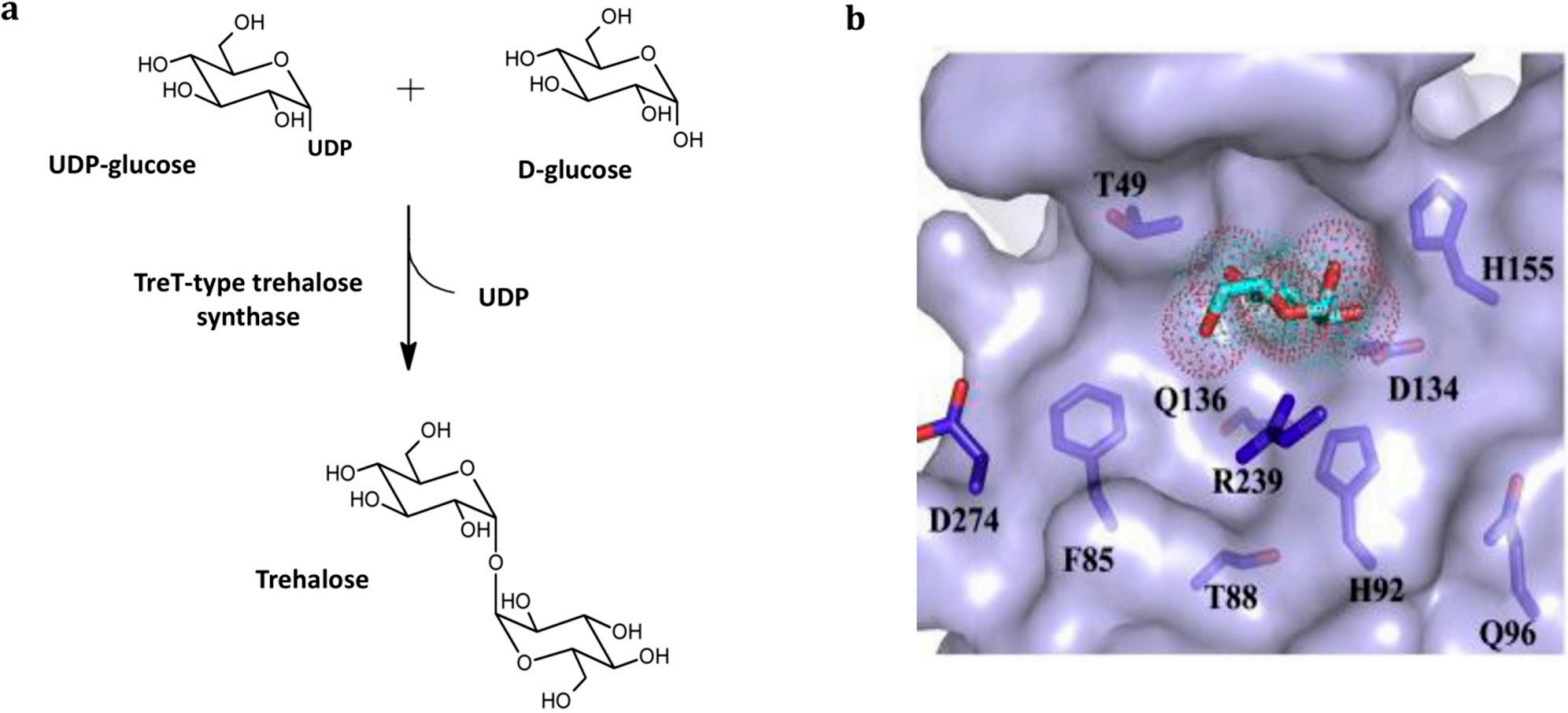
TreT-type trehalose synthase. (**a**) Natural reaction in trehalose synthesis direction starting from UDP-glucose and d-glucose; (**b**) active site residues (indicated in dark purple) surrounding the d-glucose moiety (light blue) in the acceptor binding pocket of *P. horikoshii* TreT [49]. Replacing the histidine at position 92 into an alanine via site-specific mutagenesis resulted in a 10% improvement of the activity on mannose as alternative acceptor monosaccharide.

The TreT-type trehalose synthase (EC 2.4.1.245) catalyzes the production of trehalose from nucleoside diphosphate (NDP)-activated glucose, preferably UDP-glucose, as donor and d-glucose as acceptor (Figure 5a). It belongs to the class of “Leloir” glycosyltransferases, more specifically the GlycosylTransferase family 4 (GT4), known for their strict regioselectivity and high product yields [48]. TreT is a rather “young” specificity first discovered ten years ago in the hyperthermophilic archeae *Pyrococcus horikoshii* [49]. Since then, the enzyme has been identified in many other hyperthermophilic organisms, including *Thermococcus litoralis* [50], *Thermoproteus tenax* [51] and *Thermotoga maritima* [34]. These TreTs all possess an excellent thermostability, an exceptional feature that makes them highly applicable for industrial synthesis reactions that need to be performed at elevated temperatures to prevent batch contaminations.

The TreTs from these organisms all have a broad acceptor monosaccharide specificity in common, turning them into interesting tools for analogues production [34,42]. For example, wild-type *P. horikoshii* TreT shows excellent activity on the alternative acceptors d-galactose (90%), d-mannose (80%) and even d-fructose (20%) in addition to the natural acceptor d-glucose. The wild-type *T. maritima* TreT, in turn, showed 70% and 35% conversion of d-mannose and d-fructose, respectively, into the corresponding trehalose analogues [7]. Furthermore, the acceptor promiscuity naturally present in these types of enzymes is prone to further enhancement via enzyme engineering. The activity on d-mannose of the *P. horikoshii* TreT could be improved to 90% by replacing the histidine residue at position 92 in the acceptor-binding pocket by an alanine via site-specific mutagenesis [49] (Figure 5b). Theoretically, it should therefore be feasible to produce a broad variety of trehalose analogues from the respective monosaccharide and NDP-glucose with other natural or engineered TreTs.

Recently, Urbanek and coworkers [6] exploited the potential of combining chemical synthesis with TreT-catalyzed synthesis as alternative pathway for trehalose analogue production. With the promiscuous TreT from *T. tenax*, they managed to synthesize a broad variety of trehalose analogues with high yields (up to 99%) starting from chemically synthesized glucose analogues, bearing fluoro-, deoxy-, thio- and azido-modifications. With some further process optimization and scale-up, this combinatorial approach has the potential to provide easy access to a variety of novel trehalose analogues on industrial scale.

The major drawback of analogues production via “Leloir” glycosyltransferases, however, is their need for expensive nucleotide-activated donor substrates, like UDP-glucose or UDP-galactose. To overcome this price burden, one-pot biocatalytic systems were developed that can ensure a continuous recycling of the donor substrate *in situ* through coupling with sucrose synthase or UDP-glucose-4′-epimerase [52,53]. In the past, similar coupling systems also proved very effective in enhancing glycosylation yields by other Leloir glycosyltransferases [54,55].

### 3.3. Inverting Trehalose Phosphorylase-Catalyzed Production

A third option for trehalose analogue synthesis relies on the enzymatic activity of trehalose phosphorylases (TP), present in several bacteria, yeasts and fungi. TPs catalyze the reversible phosphorolysis of trehalose to d-glucose-1-phosphate (Glc-1P) and d-glucose [56]. As the former product possesses a high energy content, the reaction can be efficiently reversed for the synthesis of trehalose and its analogues. From all known phosphorylase specificities to date, TP is the only one that has evolved independently into two mechanistically different classes, *i.e.*, retaining or inverting enzymes, leading to the formation of either α- or β-Glc-1P, respectively (Figure 3e,f).

So far, most of the work has been done with inverting TPs (EC 2.4.1.64), which belong to the Glycoside Hydrolase family 65 (GH65), along with maltose and kojibiose phosphorylase as other important members. This specificity was first discovered in the algae *Euglena gracilis* [57] and has since then been reported in both fungi and bacteria, including *Micrococcus varians* [58], *Thermoanaerobacter brockii* [59], *Bacillus stearothermophilus* [60], *Paenibacillus* [61] and *Caldanaerobacter subterraneus* [62]. In addition, a BlastP search revealed the presence of homologous genes in a broad range of other bacteria from a variety of phyla including Proteobacteria, Actinobacteria, Bacteroidetes, Firmicutes, Cyanobacteria and Green sulfur bacteria [63]. Similar to the TreT-type synthases, the inverting TPs from *B. stearothermophilus*, *T. brockii* and *C. subterraneus* were also found to be thermostable with activity optima around 75, 70 and 80 °C, respectively [60,62,64].

The inverting TPs from *T. brockii* [42] and *C. subterraneus* [62] were found to be quite promiscuous for their acceptor substrate, showing activity towards a broad range of monosaccharides including d-galactose, d-xylose, d-fucose, d-mannose, aminosugars, l-sugars like l-arabinose and l-fucose, and 1,6-linked disaccharides like melibiose or gentiobiose [42,62]. Therefore, these enzymes form useful biocatalysts for the synthesis of a variety of trehalose analogues [42] or novel non-reducing derivatives like 6-*O*-α-d-galactopyranosyl trehalose [59]. Such promiscuity is not common in all inverting TPs. The first discovered TP from *Euglena gracilis* shows a much stricter acceptor specificity with only 6-deoxyglucose and d-xylose as allowed substitute monosaccharides for d-glucose [65].

**Figure 6:**
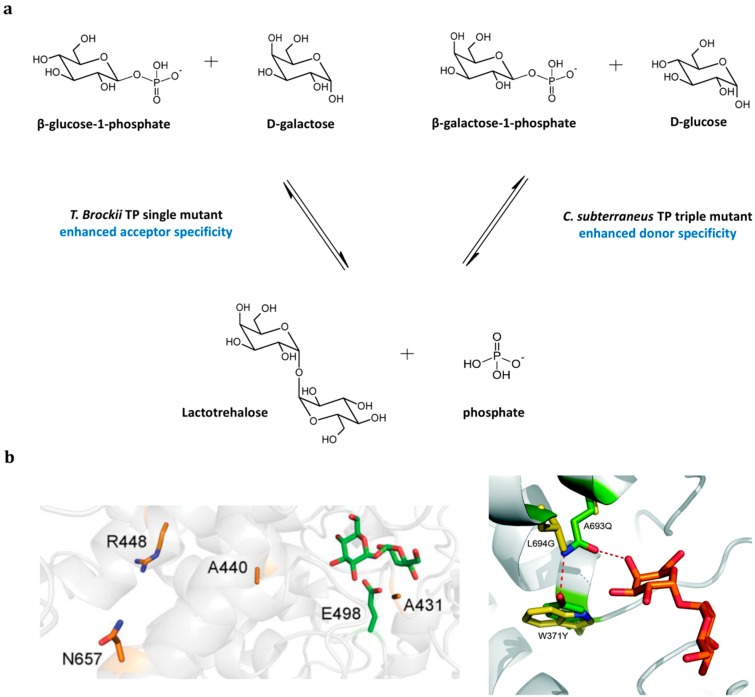
Enhancing acceptor and donor specificity of *T. brockii* trehalose phosphorylases (TP) and *C. subterraneus* TP via random mutagenesis and rational design, respectively. (**a**) Reversible conversions of β-Glc-1P with d-galactose and β-Gal-1P with d-glucose into lactotrehalose, catalyzed by the *T. brockii* TP-single mutant (R448S) with enhanced acceptor specificity, and the *C. subterraneus* TP-triple mutant (L694G/A693Q/W371Y) with enhanced donor specificity, respectively; (**b**) Active sites of *T. brockii* TP (**left**) and *C. subterraneus* TP (**right**) with the positions subjected to mutagenesis indicated. For *T. brockii* TP, the mutated R448-residue is shown in orange/blue amongst other mutated residues from the same study, and the trehalose substrate with the catalytic residue (E498) in green. In the *C. subterraneus* TP active site, the three mutagenized positions (L694G/A693Q/W371Y) causing a switch in donor specificity are indicated in green with the corresponding wild-type residues shown in yellow. The lactotrehalose substrate is indicated in orange [66,67].

Similar as for the TreT-type trehalose synthases, the acceptor specificity of TPs can be enhanced via enzyme engineering. Recently, an efficient process was reported for the synthesis of lactotrehalose from galactose and β-Glc-1P that relied on an engineered *T. brockii* TP variant with improved galactose specificity (Figure 6) [66]. By random mutagenesis, an R448S mutant was generated with a two- to three-fold lower *K*_m_-value for galactose and an improved catalytic efficiency for mannose and fructose. The latter observation ultimately suggests some involvement of the mutation in acceptor specificity.

In addition to focusing on the acceptor flexibility, engineering the donor specificity of an inverted TP could further extend the pool of trehalose analogues that could be produced with these enzymes. To give an idea of the potential, the donor specificity of the *C. subterraneus* TP could be modified towards β-Gal-1P by introducing three mutations (L694G/A693Q/W371Y), identified through iterative saturation (Figure 6) [67]. The triple mutant displayed a 10-fold improved catalytic efficiency on the alternative donor substrate, and could thus be used for the synthesis of lactotrehalose, with d-glucose as acceptor, and even galactotrehalose, with d-galactose as acceptor. Interestingly, the combined action of the *T. brockii* and the *C. subterraneus* variants can also be used for the net phosphorylation of d-galactose, which then enters such a two-step process from the acceptor side, but leaves from the donor side with lactotrehalose as intermediate (Figure 6) [67].

### 3.4. Retaining Trehalose Phosphorylase-Catalyzed Production

The main factor hindering the economic exploitation of inverting TPs for synthetic purposes is the high price of their glycosyl donor β-Glc-1P, *i.e.*, about $60 per mg. Moreover, it is not available in large amounts [68]. In contrast, α-Glc-1P is more than 2000 times cheaper (about $25 per g) and, additionally, is more amenable to large-scale production [46]. That is why, lately, interest in retaining TPs (EC 2.4.1.231) as potential alternatives for analogues production has increased. Retaining TPs are the only glycoside phosphorylases classified within a glycosyltransferase family, *i.e.*, GT4-family, and their mechanism is still a matter of debate [69]. They are mainly found in the mycelium or fruit body of fungi such as *Agaricus bisporus* [70], *Schizophyllum commune* [71] or *Grifola frondosa* [72]. In general, retaining TPs are quite specific for α-Glc-1P and d-glucose in the synthesis direction and for trehalose in the breakdown direction [71,72]. Enhancing their acceptor and donor specificity via enzyme engineering is therefore desired to broaden the spectrum of trehalose analogues that could be produced. In a preliminary study, the wild-type *G. frondosa* TP already showed minor activity on d-mannose (around 3%) as alternative acceptor substrate [73], suggesting a potential for future improvement of its acceptor specificity via rational design.

At the moment, however, the retaining TPs poor stability and expression yields are major hurdles that slow down engineering efforts and, consequently, their potential industrial application. For example, *G. frondosa* TP is stable up to 32.5 °C in the trehalose synthesis direction with activities that rapidly decrease at higher temperatures [74]. Incubation at 37 °C for 15 min in the absence of stabilizing substrates almost completely inactivates this enzyme with less than 4% residual activity left [73]. The purified *S. commune* enzyme even loses activity within a few hours when kept at 4 °C in a buffered saline solution [75]. Despite the marked stabilization effect, employing glycerol as protectant during storage could be questioned as even small amounts (10% to 20% (*w*/*v*)) of this compound induce TP-inhibition [76]. These hurdles need to be overcome in the future in order for effective industrial synthesis with retaining TPs to become feasible.

## 4. Conclusions, Outlook and Future Perspectives

It is clear that the functional oligosaccharide market has shown an impressive growth recently, not least due to the success of trehalose. As new production methods have reduced its price almost a 100-fold, myriad applications in food, biotechnological, pharmaceutical and cosmetic could be developed. Analogues of this compound are even more promising as it is very likely that they retain their stabilizing properties, and offer some additional benefits. Trehalose analogues might be interesting as low-calorie prebiotic compounds or therapeutic compounds able to reach higher systemic concentrations.

This review aimed to compare different enzymatic routes towards the trehalose analogue synthesis, of which trehalose phosphorylases and TreT-type trehalose synthases are the most promising. A combination of “smart” *in silico* based enzyme engineering to further change the specificity of these biocatalysts towards alternative donor or acceptor molecules and reaction engineering should eventually result in an industrially viable production process. Optimizing synthetic processes via coupling of reactions catalyzed by natural or engineered biocatalysts could allow for recycling of glycosyl donors, resulting in cost-effective and high-yielding analogues production. Indeed, α-Glc-1P can be synthesized from sucrose and starch by sucrose phosphorylase (EC 2.4.1.7) or maltodextrin phosphorylase (EC 2.4.1.1), whereas β-Glc-1P can be generated from maltose by maltose phosphorylase (EC 2.4.1.8). By combining one of these activities with trehalose phosphorylase in a one-pot setup, trehalose analogues can be synthesized in a very cost-effective way from cheap and abundantly available substrates with *in situ* phosphate-recycling [38,74].

Finally, it should be noted that the enzymatic conversion is only part of the story, since the downstream processing of the corresponding reaction mixtures is also a major challenge. To date, activated carbon and ion-exchange chromatography have been proposed for that purpose [42,77], but both methods suffer from scalability issues or elevated operational costs, prohibiting the scale-up of analogues purification. It is clear that the way forward in cost-effective biocatalytic production of trehalose analogues consist of the integration of enzyme and process engineering, not only aiming for the most efficient biocatalyst but also for more practical and operational procedures.
